# Interpretive policy analysis: Marshallese COFA migrants and the Affordable Care Act

**DOI:** 10.1186/s12939-016-0381-1

**Published:** 2016-06-11

**Authors:** Pearl Anna McElfish, Rachel S. Purvis, Gregory G. Maskarinec, Williamina Ioanna Bing, Christopher J. Jacob, Mandy Ritok-Lakien, Jellesen Rubon-Chutaro, Sharlynn Lang, Sammie Mamis, Sheldon Riklon

**Affiliations:** Office of Community Health and Research, University of Arkansas for Medical Sciences Northwest, 1125 N. College Ave, Fayetteville, AR 72703 USA; Department of Family Medicine and Community Health, University of Hawaii at Manoa, 95-390 Kuahelani Ave, Mililani, HI 96789 USA

**Keywords:** Health disparities, Community-based participatory research, Pacific Islanders, Health policy, Minority health

## Abstract

**Background:**

Since the enactment of the Affordable Care Act (ACA), the rate of uninsured in the United States has declined significantly. However, not all legal residents have benefited equally. As part of a community-based participatory research (CBPR) partnership with the Marshallese community, an interpretative policy analysis research project was conducted to document Marshallese Compact of Free Association (COFA) migrants’ understanding and experiences regarding the ACA and related health policies. This article is structured to allow the voice of Marshallese COFA migrants to explain their understanding and interpretation of the ACA and related polices on their health in their own words.

**Methods:**

Qualitative data was collected from 48 participants in five focus groups conducted at the local community center and three individual interviews for those unable to attend the focus groups. Marshallese community co-investigators participated throughout the research and writing process to ensure that cultural context and nuances in meaning were accurately captured and presented. Community co-investigators assisted with the development of the semi-structured interview guide, facilitated focus groups, and participated in qualitative data analysis.

**Results:**

Content analysis revealed six consistent themes across all focus groups and individual interviews that include: understanding, experiences, effect on health, relational/historical lenses, economic contribution, and pleas. Working with Marshallese community co-investigators, we selected quotations that most represented the participants’ collective experiences. The Marshallese view the ACA and their lack of coverage as part of the broader relationship between the Republic of the Marshall Islands (RMI) and the United States. The Marshallese state that they have honored the COFA relationship, and they believe the United States is failing to meet its obligations of care and support outlined in the COFA.

**Conclusion:**

While the ACA and Medicaid Expansion have reduced the national uninsured rate, Marshallese COFA migrants have not benefited equally from this policy. The lack of healthcare coverage for the Marshallese COFA migrants exacerbates the health disparities this underserved population faces. This article is an important contribution to researchers because it presents the Marshallese’s interpretation of the policy, which will help inform policy makers that are working to improve Marshallese COFA migrant health.

## Background

The Marshallese population is rapidly expanding in the United States, having more than tripled between 2000 and 2010 [1]. The United States controlled the Republic of the Marshall Islands (RMI) as part of the Trust Territory of the Pacific Islands (TTPI) from 1947 to 1986. Upon the signing of the Compact of Free Association (COFA) between the RMI and the United States in 1986, the RMI became a sovereign nation. The COFA allows Marshallese citizens to lawfully enter the United States, and to reside, work, and study without a visa or permanent resident card [2]. Based upon local health department and school records reported by the RMI consulate in personal communications with the lead investigator, an estimated 10,000 Marshallese people currently reside in Arkansas, the largest population of Marshallese living in the continental United States [3]. Beginning with only a few Marshallese migrants arriving in the late 1980s to work in the poultry industry in northwest Arkansas, the Marshallese community has grown steadily over the past three decades [4]. Compared with the general U.S. population, Marshallese migrants are typically younger, have less educational attainment, and higher rates of poverty [5].

The RMI was the principal site of the United States’ nuclear testing program from 1946 to 1958 [6, 7]. The burden of these nuclear tests were equivalent in payload to more than 7,000 Hiroshima-sized bombs, and the Marshall Islands are now considered to have the highest level of nuclear contamination in the world [7]. The nuclear tests destroyed entire atolls in the island chain and contaminated the plant and sea life of many other islands [7–10]. The nuclear explosions, subsequent contamination of the Marshall Islands, and the relocation of Marshall Islanders permanently altered the traditional diet and lifestyle of the Marshallese, and the resulting changes in their diet has serious health effects [7, 11–14]. The Marshallese population living in the RMI and the United States face significant health disparities [15–18]. Rates of diabetes are documented at more than 400 % the national average [19]. Infectious diseases, particularly hepatitis B, tuberculosis (TB), and Hansen’s disease (leprosy) are also found at higher rates among the Marshallese than in the general population [20–27]. In addition, Marshallese mothers in the United States give birth to low birth weight babies at higher rates than the general U.S. population [28].

### Health care reform policy and its impact on Marshallese health

The Patient Protection and Affordable Care Act (ACA) was signed into law by President Obama in March 2010 and later upheld by the Supreme Court in June 2015 [29]. The law creates marketplaces where consumers can purchase subsidized health insurance, and it also requires legal residents to obtain health insurance [30]. The ACA offers states the option to expand Medicaid to more low-income (133 % of poverty level) residents. Arkansas is one of 29 states that expanded Medicaid to low-income adults [31, 32]. Nationally, these programs reduced the uninsured rate by 3.5 %, from 17.3 to 13.8 %, and in Arkansas, the uninsured rate declined from 22.5 to 11.4 % from 2013 to 2014 [33]. However, this public policy has not benefitted all. Marshallese COFA migrants living in the United States have limited access to federal and state benefits programs under the ACA [5, 34–37].

To implement the ACA, the state of Arkansas employed navigators and in-person assistors (IPAs) to help consumers understand insurance options, determine eligibility, and facilitate enrollment [38]. In northwest Arkansas, three bilingual native Marshallese IPAs and navigators were hired to work with the Arkansas Department of Health and the local legal aid office. Only one navigator was located within the community where most Marshallese live, and the two IPAs were located in an adjacent town. The IPAs were not allowed to process applications offsite. Thousands of Marshallese attempted to sign up for health insurance, with each new application taking approximately two hours; many of the applications also required additional follow-up.

As lawfully present migrants, Marshallese COFA migrants are required to purchase health insurance, are eligible for advanced premium tax credit subsidies, and are subject to the standard penalties if they do not enroll in a health plan [39]. However, COFA migrants are not eligible for Medicaid or Medicaid Expansion [37]. When the COFA was signed in 1986, Marshallese migrants were eligible for Medicaid and other federal safety net programs. In 1996, however, COFA migrants living in the United States were rendered ineligible for Medicaid with the implementation of the federal Personal Responsibility and Work Opportunity Reconciliation Act (PRWORA). Under PRWORA, COFA migrants are excluded from the category of “qualified immigrants” eligible for Medicaid [35, 40, 41]. While PRWORA disqualified COFA migrants from eligibility for these federally-funded benefits programs, state governments have the discretion to continue Medicaid coverage exclusively with state funds [36]. Arkansas has not funded Medicaid for COFA migrants [34, 35, 37].

The lead author began working with the Marshallese community in early 2013 to address type 2 diabetes [13]. However, in every community stakeholder meeting, more than 30 meetings in total, the ACA and Medicaid Expansion were brought up as major concerns. Honoring our commitment to ensure the community is driving our research agenda, we chose to conduct interpretative policy analysis research to document Marshallese COFA migrants’ understanding and experiences regarding the ACA and related health policies. This article is structured to allow the voice of Marshallese COFA migrants to explain their understanding and interpretation of the ACA and related polices on their health in their own words. In addition, we offer policy recommendations to address participant concerns.

## Methods

A qualitative design was used as an exploratory method to better understand how the Marshallese interpret the ACA and related health policies, as well as how the Marshallese describe the effect of these policies on their lives [42]. The guiding research questions are: For Marshallese living in the United States, 1) what is their understanding of and what are their experiences with the ACA and related health policies? and 2) what effect do the ACA and related health policies have on the community’s health? A semi-structured interview guide was created with open-ended questions to allow participants to speak freely and give in-depth responses about their understanding and experiences, while also ensuring all focus groups and individual interviews covered the same topics [43]. The interview guide was developed with input from our community-based participatory research (CBPR) stakeholders.

Participants were recruited through our CBPR partnership with the local Marshallese community. Participants were 18 years of age or older who self-reported as Marshallese. Community members who met the participation criteria were invited to take part in the study via e-mail, church groups, and Facebook. Participants were given the opportunity to provide verbal consent. After consent, participants completed a brief survey that captured demographic information, insurance status, and whether or not the participant had a primary care provider. Following the brief survey, five focus groups were conducted at a local community center. Three individual interviews were conducted with people who were unable to attend the focus groups, in locations of their choice [44]. Bilingual research staff helped conduct each focus group and individual interview. Qualitative data were collected from 48 participants. Participants were given a $20 gift card as remuneration for their contribution. The Institutional Review Boards (IRB) at the University of Arkansas for Medical Sciences and the University of Arkansas at Fayetteville reviewed and approved the study procedures.

Focus groups and individual interviews were recorded and transcribed verbatim. Data collected in the Marshallese language was first transcribed in Marshallese and then translated into English. The transcript was confirmed by a second Marshallese translator prior to coding. Content analysis was performed. The researchers coded the data for a priori themes (from the interview guide) as well as emergent themes. Both a priori and emergent themes were organized into a codebook, which two qualitative researchers used to code the transcripts. A summary of themes was presented and discussed with the CBPR team, including seven Marshallese community co-investigators. The Marshallese co-investigators participated throughout the research and writing process to ensure that cultural context and nuances in meaning were accurately presented. In analyzing our transcripts, we reached saturation with thematic codes after the first two focus group interviews. The remaining interviews continued to provide the same themes, but with additional richness. Themes were consistent across both the focus groups and the individual interviews. Working with the Marshallese community co-investigators, we selected quotations that most represented the participants’ collective experiences. The quotations selected were obtained from 21 separate participants from all five of the focus groups and three individual interviews.

## Results

### Participant demographics

Table 1 presents the information related to participants’ ages, income, and health insurance coverage; percentages reported below are based upon the number of participants who responded to each item. The majority of the 48 participants (89.1 %) reported income at or less than $30,000. Twenty-five (25) participants (54.3 %) reported having health insurance and 21 participants (45.7 %) reported having a primary doctor to meet their family’s health care needs. Interestingly, of the 25 people with insurance, only 15 of them have a primary care physician; and of the 21 people with a primary care physician, 15 have insurance coverage.

**Table 1:** Participants: Age, Income, and Health Insurance Coverage

Response Category	(*N* = 48)	Percent of sample^a^
Age		
18–24 years of age	3	6.5
25–30 years of age	4	8.7
31–40 years of age	9	19.6
41–50 years of age	17	37.0
51–60 years of age	9	19.6
61–70 years of age	4	8.7
71 years of age and above	0	0.0
Annual Income		
Below $10,000	17	37.0
$10,000–$20,000	10	21.7
$20,000–$30,000	14	30.4
$30,000–$40,000	3	6.5
$40,000–$50,000	2	4.3
Do you have health insurance?		
Yes	25	54.3
No	21	45.7
Do you have a primary doctor for your family’s health needs?		
Yes	21	45.7
No	25	54.3

^a^Percentages are based on the number of responses for each item

### Themes and sub-themes

The content analysis revealed six primary themes (three a priori and three emergent). These themes and sub-themes are outlined in Table 2. Results are organized within these themes.

**Table 2:** Themes and Sub-themes

A Priori Themes	*Sub-themes*
1) Understanding. Participants’ understanding of the ACA and related policies	*In-depth understanding* *Lack of understanding* *Lack of understanding because of poor follow up* *Lack of understanding about insurance premiums, co-pays, and who accepts their insurance* *Lack of understanding and frustration about tax penalties*
2) Experience. Participants’ experiences with ACA and related policies	*Some get approved and some do not* *The ACA is not affordable* *Improving the experience*
3) Effect. Participants’ description of how the ACA and related policies’ effect participants/community health	*Health status* *Treatment differences*
Emergent Themes	*Sub-themes*
4) Relational/Historical Lenses. Participants’ view of the policies in relation to the Compact of Free Association, U.S. nuclear testing, use of their land, and the current relationship with the U.S. military	*Friendship agreement* *Nuclear testing* *Value of land* *Military Service*
5) Economic Contribution. Participants’ view of the policies in relation to participant contributions to state and federal taxes and the local economy	
6) Plea. Participants’ discuss their desire to have their voices and experiences heard and their culture’s method of advocacy	*Hear our voices* *Culture and advocacy* *Good friends*

### Understanding

#### In-depth understanding

Many participants had an in-depth understanding of the ACA and were able to describe the program in great detail. Participants stated the ACA was established to help those without insurance obtain health care through primary care physicians. The majority of participants also clearly understood the ACA required them to purchase insurance and they would face financial penalties if they did not. The in-depth understanding is illustrated in the following quotation.

The reason this law was created by Obama, was so that every one of us who are beneath the poverty level and little bit higher can enroll in it … That’s what I know from what I’ve heard. [ACA] is to make those who are under the poverty level in America, as well as those who cannot afford it, to be able to afford to purchase health insurance, to be able [to] prevent all the diseases they encounter in the United States. Also, to make it affordable for those who were going through hardships and that when they get sick, they weren’t able to get [any] insurance due to some sort of pre-existing conditions or diseases that [they] had been diagnosed with.

#### Lack of understanding

While many participants were quite knowledgeable about the ACA, others reported significant confusion and lack of understanding. Participants explained many Marshallese, in particular recent migrants or elders, were unsure what the ACA was and unsure of their eligibility for insurance coverage through the ACA. “I see that there are many Marshallese who are still confused.” Other participants stated that they did not fully understand the ACA. “[The] people who help us apply sometimes explain to us what the Obama Care is all about, but they don’t fully explain, so sometimes we are still lost.”

Participants repeatedly stated they wanted to understand and actively sought out information from IPAs, navigators, and other health professionals. Although many tried to inquire about the ACA and how it affected them personally, participants state that they received conflicting information regarding their eligibility for insurance through the ACA and the penalties they faced if they did not obtain insurance coverage.

Whenever we ask [the federally qualified health center located at the local public school] if we can apply [for insurance] or if there is any assistance, they tell us to call the DHS [Department of Human Services]. When we call and check with them they say we’re not eligible. But what is this that we’re hearing about the Obama Care?

Participants explained that they were told they were not eligible due to their COFA migrant status and lack of U.S. citizenship. “When I went to DHS they told us that we are not [U.S.] citizen.”

Several participants did not understand why they were left out of Medicaid Expansion and inquired, “Is there anyone that can explain to me why am I am not qualified for Medicaid?” Of the three Marshallese IPAs and navigators hired to assist with applications, only one of them was located in the Marshallese community. As a result, many participants reported trying to use the phone assistance center or non-Marshallese staff to assist them with their application. Participants observed a broad lack of understanding among non-Marshallese staff about their COFA migrant status and eligibility for the ACA and Medicaid. Participants expressed frustration because ACA staff did not understand their COFA status or even where the Marshall Islands are located, “You drop your bombs on our land and you don’t even know where the Marshall Islands are?” This lack of understanding among ACA staff meant those trying to sign up were often sent to speak to someone else without being helped. Participants described being referred elsewhere five or six times before finding someone who could help them, and many reported giving up before completing their applications.

#### Lack of understanding because of poor follow-up

The participants also explained they had reduced understanding and frustration because of the lack of follow-up after applying for the ACA. Participants reported filling out forms multiple times and calling the information line two or three times per week for a year without a resolution of their application. Participants stated that after several calls “no one can help you, you just give up.” The lack of follow up was a common experience with the application process and a frustration voiced by many participants.

The way I see it it’s [ACA] good, and they [the IPAs] are helpful in many ways, but for me, I lose interest in doing anything about it. In another word, it was a waste of my time and effort. I filled out applications and go here and there and trying to do what I can to be insured. Today, I still don’t have insurance and so doesn’t my family. [IPAs] come and work with me and my family and it’s also a waste of your time. I gave you all my information and still I haven’t heard from any one and I still don’t know my status. Today, I still don’t know my application status and still uninsured.

They tell us to fill out the forms. And then what… because I already filled out my form and I already sent it. Now they told me to apply for the Obama Care. I did and when [DHS] looked over it, they saw that I’ve apply two times so when I call the people for Medicaid, they told us to call back, and when we do, they don’t answer. I don’t know, maybe they lied to me.

#### Lack of understanding about insurance premiums, co-pays, enrollment periods, and who accepts their insurance

Participants explained that the U.S. health care system, with its insurance premiums, open enrollment periods, co-pays, and primary care providers, is very different than the health care system in the RMI and is often confusing to Marshallese migrants. “There are those who don’t understand [the U.S. health care system]. From point one and all the way through and still don’t understand....they don’t understand.” Participants noted a significant lack of understanding and widespread confusion about the cost of insurance premiums and co-pays. “Some say that if you have the Obama Care, if you have one [insurance card] you will not have to pay for your hospital bills....if you have Obama Care your hospital visits will be free.” Participants continued to describe the lack of understanding surrounding co-pays as the following quote shows.

It [ACA] was for everyone who weren’t able to afford it before. That those with low income can be qualify for it [ACA] and then they don’t have to pay for the medical costs. Like they won’t owe anything, just like it is free. But nowadays, what I am hearing is, those that were enrolled in this program still owe money or they still have medical bills. So, this is how much I know.

Participants also noted pervasive confusion about open enrollment, “The first one says one-time deal. It’s a one-time deal. But now, we heard that they reopened the door for another open enrollment for the Affordable Care Act.” Another noted, “One day, I was watching TV and I heard the news was saying that enrollment period was over… it was the deadline and no more enrollment. I haven’t heard anything else since then.” Some participants were also confused about the level of coverage that is provided and if all health care providers accepted the insurance obtained through the ACA: “I hear people say that Obama Care are accepted at some hospitals and not [at] other hospitals.” Another stated, “They said some hospitals accepts, and some they don’t. It’s not good at all.”

#### Lack of understanding and frustration about the tax penalty

Participants discussed at length their confusion and frustration related to the tax penalty for not having insurance. Participants explained that the tax penalty is a top concern and one that is most discussed in the Marshallese community.

One of the scariest thing … now they’re saying that if you don’t… make payment, they will charge you thousands. And they’re talking about the income tax… they said if you didn’t get any insurance, if you didn’t apply for insurance and you don’t have any insurance and if you have money in your bank account, they will garnish it. I don’t know where this is coming from, but these are the rumors that have most of these people confused.

### Experiences

#### Some get approved and some do not

Participants were the most vocal about their confusion and frustration with the lack of consistent experiences regarding who is and who is not approved for Medicaid Expansion. As one participant explained, “When we apply for [Medicaid] some get in and some don’t.” The experiences differed widely: “I was told to wait to receive my eligibility letter, which I have never received.” Another participant recounted, “Our older siblings, we took them, and helped them get insurance, but no luck. Some people get approved and some they don’t get approved.” Even among those who had received coverage there were inconsistent messages. “I’ve been told that I wouldn’t qualify for Medicaid, but next thing I know I received my approval letter.” Another participant stated they received an insurance card, but later found that the card was not accepted, as the following quote illuminates.

The card was not recognizable. When we visit a doctor the clinic says the cards are not acceptable, and then when ask why and are told that it is because we are not citizens, so the question is, why were we given insurance and the cards in the first place? Why approve our status during the process and then later tell us during our visits to the doctors ‘you’re not approved’ because you’re not a citizen? What’s worse is that after they have collected all our personal information and our social security numbers, date of births they tell us they are not approved because of our status. I’ve tried many times to apply and I just gave up. I don’t want to try anymore.

The participants also discussed their attempts to try and understand why some were approved and some were not, but they could not see any pattern.

When we go with [name removed] we don’t get in Obama Care, but some people do. Like I already said, we filled out forms, but we didn’t get anything. All the people that filled out forms said that they didn’t get anything either, but the preacher and his family got their [insurance]. Their kids have food stamp. See! But, they were born in the Marshall Islands.

Another participant added, “That’s what I said. Why do some get approved, and others don’t.” Interviewees also described how even within one family some people were approved and others were denied. “I would like to say that after I applied my family was approved, except my wife, and so I would like to know why she was denied.”

#### The ACA is not affordable

When discussing their experiences with the ACA, many voiced their concern that the tax credit alone, without access to Medicaid Expansion, meant that the ACA was not really affordable for them. “You said Affordable Care Act, but I cannot afford it.” Participants explained most Marshallese migrants are low-wage workers in the poultry industry and one worker is often supporting a large household of children and elderly family members. Providing insurance coverage for all in their household is not obtainable. Another participant expressed concern with their family members’ ability to pay for the insurance, stating that “Even with the Premium Tax Credit, it is [too] expensive for them to afford it.” Other interviewees expressed similar opinions. “My budget can no longer afford it [insurance]. For most of us, there will be only one breadwinner but we take care of everyone, even extended family members that live with us.”

I do have issues with this. They [the Marshallese elders] have no income, but they’re only eligible for the Premium Tax Credit and aren’t eligible for Medicaid. Their children only make so much; maybe let’s say $17,000 annually for many people in the household. They don’t make that much annually, but the credit they received from federal [government] is less comparing to what they need for their health. Why enrolling these Marshallese in the Marketplace when they’re not eligible to these [Medicaid Expansion] assistance?

In addition, participants expressed concern that even after they signed up and were approved for insurance that if they were not able to afford premiums for one month, then they would be dropped from insurance and have a lapse in coverage.

#### Improving the experience

Despite their experiences, participants were generally supportive of the ACA, but felt that the system needed improvement. “I’m a big fan. I’ve seen a lot of technical issues when it comes to the actual process of the enrollment. The overall concept really makes sense, but when it comes to execution, there’s a huge gap there and a big barrier.” One participant continued, “The system needs to be user friendly. [It] is just way too much. We need to make it user friendly. I think the concept of Affordable Care Act is awesome. Execution, poor execution. We need to come up with better ways.”

### Effect

#### Health status

Participants discussed how the absence of insurance coverage impacts their health. Participants stated, without insurance, many Marshallese people do not seek health care services or fill their prescriptions due to the cost. “For me, sometimes when I’m sick, I don’t go seek medical attention, because I can’t afford to go. So, I just stayed home and do nothing. And I know it’s not healthy.”

For me, it’s not good because for someone diagnosed with diabetes, sometime I don’t take my medication as I’m suppose because I can’t afford to buy [the medication]. Because I’m not working, and it’s hard to stay healthy when there is little resources to get the help we need. It’s just not healthy.

#### Treatment differences

In addition to not being able to afford health care services, participants shared their perceptions of discrimination based on insurance status, which they believe results in less effective or different treatment from health care providers. “They don’t care about us—the patient—because we don’t have insurance.” Participants described their experiences with perceived discrimination due to their insurance status.

There are times when those who don’t have insurance are not cared for. When we go to see the doctor, they say “oh, take them out” [discharge the patient], but they’re not really feeling any better because what? They don’t have insurance? There are a lot of people who don’t have Obama Care. Our older siblings, we helped them [apply for insurance], but they haven’t gotten their insurance. But when they go visit the doctor, she had no insurance because she has not been approved. For those who don’t have insurance can be in no more than two to three days in the hospital; they don’t care, because they don’t have an insurance.

### Relational/historical lenses

#### Friendship agreement

Participants interpreted the ACA and restrictions on health insurance coverage through the lenses of the COFA, prior nuclear testing, past and current use of their land, and the RMI’s current relationship with the U.S. military. Participants clearly understood the terms of the COFA, but they interpret it as more than a legal agreement; they see it as a commitment of friendship between the RMI and the United States.

The Compact of Free Association was passed in June of 1986. It is a law that binds the friendship between the Marshall Islands and the United States. To simplify this for better understanding, this law was enacted for security purposes for using Kwajalein Atoll. This is one of [the] purposes of the COFA as well as being able to protect the Pacific Ocean. [It also] protects America from the countries that America is afraid of regarding the military and combat. We enacted the COFA so [the] U.S. can help the Marshall Islands in any way possible.

Because the United States provides most of the funding for health care in the RMI as part of the COFA, participants believed they would have the same health care access when they came to the United States.

When I first left the Marshall Islands my thoughts about visiting a doctor would be like how it’s done at home [in the Marshall Islands]. Back home we have benefits that are granted to our government from the federal government of the United States through the Compact of Free Association that makes seeking medical attention easy and affordable. When I first moved to the United States, I lived in Hawaii and I thought that I was able to seek medical care under what I understood as ‘equal protection under the law.' But, everything is different and harder here. What I don’t understand, that is mind boggling, is that back in the Marshall Islands, health care is fully funded by the U.S. government, but it is not the same when we are physically here in America. I thought since there was an agreement between my country and the United States, and they [the United States] used our lands for nuclear testing that they would help in some ways, but I guess that’s not how they do things here.

Participants referred to health care insurance coverage when the COFA was signed before welfare reform in 1996. Several participants said that the RMI had worked in good faith with the United States and that the United States had failed to fulfill their responsibility in the partnership.

Back when late President Amata Kabua was our leader [of the Marshall Islands] they [the United States and Marshall Islands] seemed to be in agreements with many things. I was still a young lad, and when I grew up and learned to read, my understanding was that there was a law stating we could seek medical attention while living in America. Like I said, I thought we would have been fully funded in the health/medical system since it is easy for us to come to the United States and also because we are fully funded in the Marshall Islands by the U.S. government, but when we move here where it truly belong to the Americans, we are otherwise funded. You would think that since we moved to their country they would help, but it seemed as if they have closed their hands and turn their heads the other direction so all we can see is the back of their heads, and not help us when we are in need.

#### Nuclear testing and the value of land

A participant summarized what many others voiced about the connection between the nuclear testing and the current restrictions related to health insurance: “What I’m thinking right now is for the Americans to acknowledge that what they did to us and our country with the testing of nuclear bombs should be recognized and take full responsible for what happened.” Other participants were much more direct in stating that the United States has a responsibility to provide health care access to Marshallese COFA migrants because of U.S. nuclear testing.

It’s okay to claim yourself as a COFA migrant; it’s okay to talk to politicians about lack of access to health care because of our status. It’s okay to tell them that you know our lands were used as grounds for nuclear testing and because of that, now we see a lot of people with health issues due to the testing, so it’s okay for us to tell them: You're wrong, you know. You can’t just give me a ticket and say I’m done with you after you destroyed our lands. You know it’s going to have to be more than that. Don’t just give me a ticket and say, I’m done, you know, because the aftermath effect of the nuclear testing is pretty profound. It caused health care issues and caused also social issues as well. So, it is quite okay to talk about it and tell your politicians that you know we’re not here because we want to be here.

I think the main thing I want is for them [Americans] to acknowledge us, know what they did to us and stop turning a blind eye, pretending that it didn’t happen, because the reason these things arises [health issues] and the reason people die young are due to diseases that occurred/resulting from the explosion [nuclear testing] that were tested on the islands. They [Marshallese people] didn’t just get sick and die. They died from being poisoned. Not just one [nuclear test] but fifty-eight or fifty-six, I couldn’t be too sure with the numbers…I know these bombs were very powerful and affected the whole Marshall Islands.

Participants explained the cultural importance of their land as “priceless.” “The most important thing, and as core to our identity, core being, legacy, and inheritance for future generations.” Participants stated that they saw the lack of coverage from the ACA’s Medicaid Expansion as a betrayal of their relationship with the United States. “We gave our best gift to the United States and you won’t even give us health care, which is a small gift in return.”

#### Military service

In addition to nuclear testing, participants stated that Marshallese serve in the U.S. military at a greater per capita rate than U.S. citizens, and they die during military service at higher rates than other U.S. citizens. Those interviewed also highlighted the current presence of the Ronald Reagan Missile Defense Base on Kwajalein Atoll in the RMI and its use for strategic U.S. military defense.

It is way more deeper than just the COFA agreement. You know we have a military base in the Marshall Islands that is positioned there, and this military base is Kwajalein Missile Range, Reagan Missile Range, and basically, this was put there as a shield to the US. Our lands are used as security shield [for] security purpose, you know, more than just a COFA, so it is okay for COFA citizen to tell them that.

We’re giving our own to the war zones, and they die. They [United States] need to really look into how our country collaborate with their country [United States] - our relationship with them [United States]. And maybe sometimes, they [Untied States] don’t comprehend it. So they [United States] can’t understand our relationships as a nation to another nation. Because for them, they have so much freedom to come to the Marshall Islands. They [United States] can come to our islands and recruit soldiers for them [US military] and sometimes these things are hard for us to do but we give our lives for them [United States].

So you guys [United States] should look out for us. The reason America is strong is us. Yes, why do you think America is strong in combat? We allowed them [Untied States] to test their nuclear weapon in the Marshall Islands. You allowed them to and still allow them to release missiles from one place to another. And, to poison Bikini Islands, not just Bikini Islands, but all of Marshall Islands.

### Economic contributions to the national and local economy

Many of the participants are frustrated that they are paying state and federal taxes, including Medicaid tax, but are excluded from Medicaid and other federal benefits. “You work here in America, you have to pay taxes to them.” “We pay taxes and pay into the Medicaid and Medicare system and yet, we cannot qualify for Medicaid.” Participants expressed high levels of frustration among Marshallese migrants who have been working and paying taxes to the United States for most of their working lives.

You deduct tax out of my salary for about 20 to 30 years and when I apply for Medicaid, [I am told] NO..... Tell me how many Marshallese are here [in the United States]. Who is suffering? Us [Marshallese] or you [United States]? Who’s benefitting? Us [Marshallese] or you [United States]?

Participants discussed their frustration that their contribution to the local and national economy were not recognized because of discrimination.

[Marshallese COFA migrants] generate revenue to the city, generates to the state, and to the federal, and now that you actually reside here, they make these our barriers or issues. You don’t qualify for this and that, you can’t because you are Marshallese. Well, I believe that is called being discriminated against.

### Plea

#### Hear our voices

The overall tone of all of the focus groups was one of frustration. Participants were frustrated that the Marshallese have given, and continue to give, their most valuable gifts of their homelands, military service, and labor to the United States, while the United States is not reciprocating. Participants want their voices to be heard in the hopes that illuminating their needs will contribute to policy changes.

By recording our voices and discussions today so that Arkansas can recognize us and open these opportunities to us. When people don’t understand, they tend not to speak up. They keep silent. Imagine how many years they’ve been working different places…and we’re paying toward Medicaid. That’s why we represent, as our saying goes, *Jepilpilin ke ejukaan* (interpreted as ‘accomplishment through joint effort’). We’re here now and we’re the voice of the community. And by voicing our discussions through the recorder, it will show our issues.

#### Culture and advocacy

Participants discussed the lack of attention from the United States on the Marshallese need for health care coverage. When asked about the absence of advocacy within the Marshallese community, participants explained advocacy was a foreign concept to their culture.

We don’t normally just speak out because we have too much respect. It’s a culture [value]. And, when I say we don’t normally argue, it’s because it’s our culture. We usually appreciate people and say, she won’t argue because she have too much respect, she won’t show off her powers, she won’t show off her strength, all because she grew up respecting her culture. And so, when she moves to the United States, she doesn’t voice her concern because she was brought up to respect the culture and others.

#### Good friends

When participants talked about their exclusion from Medicaid and Medicaid Expansion that is part of the ACA, they did not discuss advocating for policy changes. Instead, participants continually noted they had “been good friends” to the United States and that they expected the United States to be good friends in return. The Marshallese culture is one based on a system of reciprocity and trust, rather than advocacy or confrontation. As one participant stated, the COFA “is a law that binds the friendship between the Marshall Islands and the United States.”

At the end of one of the focus groups, a Marshallese pastor prayed with participants for their voices to be heard and that unjust laws would be addressed. His prayer articulated the desires of the community to improve the health of the Marshallese living in Arkansas.

Our heavenly Father, we praise thee and worship you. We want to thank you for a fellowship in which there were accomplishments that were needed to reveal to make it better for the Marshallese, those who are dwelling in this community. We hope that it can also work for other communities in other states- make it better, the hardships that these people are facing. Lord, we ask that you open doors. Let your favor take place. There will be laws and magistrates that will make it unbearable for us, but we put our faith in the God we know, who lives amongst us. There will be miracles that have to take place for these hardships to lift off of us. These we pray. The purpose for this fellowship, O Lord, is so you can combine all these thoughts for the betterment.

## Discussion

The ACA and Medicaid Expansion provide health insurance coverage for many Americans and has drastically reduced the number of uninsured. However, Marshallese COFA migrants have not benefited equally. There are a few essays that describe the lack of access to Medicaid and insurance coverage for COFA migrants [2, 19, 37, 45, 46]; however, no previous research presents the COFA migrants’ understanding of and experience with the ACA or related health policies. This article adds an important contribution to the literature, as it presents the Marshallese’s interpretation of the policy in their own words.

Marshallese COFA migrants are caught in a broken and unjust system. By law they are required to have insurance and pay state and federal tax; however, they are excluded from Medicaid and Medicaid Expansion offered under the ACA. This exclusion significantly impacts the health of the lowest-income Marshallese migrants who are below, or near, the poverty line and struggle to support their multi-generational households. While COFA migrants are eligible for tax credits to assist with insurance coverage, these tax credits are based on earning above 133 % poverty. Most Marshallese fall below this level, and those under 133 % would typically qualify for Medicaid and would be exempt from paying premiums. Because COFA migrants do not qualify for Medicaid, the cost of premiums are far more expensive than they can afford. This means most Marshallese cannot afford to purchase insurance through the ACA. While they cannot afford insurance, they are still required by law to have insurance, or face stiff tax penalties.

Those Marshallese who attempt to apply for insurance under the ACA face significant barriers navigating the application process due to language differences and a lack of understanding of the policy by ACA enrollment staff. In northwest Arkansas, only three bilingual staff were hired, and two of those were not stationed in the city where most Marshallese live. Participants recounted numerous stories of unsuccessful attempts to gain insurance, yet they will be penalized if they do not comply with the law requiring them to have health insurance. Most participants’ demonstrated a high level of understanding of the ACA and details of their COFA migrant status. Their confusion and frustration centers on the lack of understanding about their eligibility among the non-Marshallese staff who are supposed to help them enroll in the ACA, coupled with frustration with the contradictory answers they receive concerning how these health care laws affect them. Marshallese COFA migrants experience extended wait times, as well as widely varying experiences with who is and who is not approved for coverage. In addition, there is evidence that the lack of insurance and long delays in approval for insurance is affecting the Marshallese health and access to health care. Participants also reported perceived discrimination when they try to access health care services.

Participants do not view the ACA and related health policies as discrete and separate issues, but instead have a more holistic view. They interpret the ACA within the much broader context of the ongoing relationship between the RMI and the United States as outlined in the COFA. Participants explained that the COFA is much more than a legal agreement between two nations. For the Marshallese, this agreement represents a compact of friendship between the American and Marshallese people. As our Marshallese community co-investigators and CBPR stakeholders explain, within the Marshallese culture the COFA agreement is more akin to a familial relationship in which friends are revered and honored. Within this relationship, each party is committed to taking care of the other’s needs. “Jeṃ-jerā” is the Marshallese term that describes how the Marshallese understand the compact relationship. The concept of “jeṃ-jerā” roughly translates as “blood brothers,” or a lasting relationship in which non-family members are placed into a deep nexus of mutual caregiving and obligations within an adoptive family network. The Marshallese honor their friendship with the United States by giving their lands for the U.S. military’s nuclear weapons testing program and missile defense program. Community co-investigators continually stress that nothing is more important to the Marshallese than their land, and they explain that “without land you are a person of no consequence.” Participants and community co-investigators feel the United States has taken the most precious gift of land from the Marshallese and then betrayed them by not providing the small gift of access to health care coverage through Medicaid Expansion.

Traditional concepts and methods of policy advocacy in the United States are antithetical to the Marshallese culture’s commitment to respect, humility, and graciousness. The undertone of the interviews reveal frustration and dismay that the United States has turned their backs on the Marshallese people and refuses to honor the friendship agreement. At the same time, participants are gracious, humble, and kind in their attempts to voice this frustration. During the interviews there was an obvious struggle between participants’ desire to voice their concern and frustration with the policies, while still behaving respectfully and graciously. Throughout data collection and fieldwork, participants and CBPR stakeholders discussed the need for health care coverage by reiterating that the Marshallese are a good friend to the United States. Rather than demanding their rights, the interviewees continually recount U.S. nuclear testing on their islands, the presence of a U.S. military instillation in the RMI, and Marshallese service in the U.S. military. Participants emphasize that since they have been good friends to the United States it is only logical that the United States honor their friendship by being a good friend in return. However, in their assessment, the “jeṃ-jerā,” or “contract of the friendship,” is not being honored by the United States.

### Limitations and strengths

One of the primary limitations of the study is that the qualitative data was collected from a convenience sample and is limited to participants in Arkansas. As Table 1 shows, we achieved a diverse sample within the Marshallese community. Participants included a range of ages, with approximately half insured and half uninsured. Similarly, approximately half had a primary care physician and half did not. While only 50 % of participants were uninsured, local studies estimate that the uninsured rate among COFA migrants in Arkansas is closer to 70 % [47]. In addition, while bilingual research staff co-facilitated interviews, all participants also spoke English. Participants’ understanding of insurance may have been increased because of their relatively high insured rate and English proficiency.

We reached saturation after the first two focus group interviews, and consistent themes emerged across the remaining focus groups and individual interviews. For internal validity and credibility, qualitative researchers are concerned with the degree to which their findings represent the lived experiences of the participants [48, 49]. The intensive level of involvement of native Marshallese community co-investigators greatly increases the internal validity of the results. Community co-investigators enhanced the data analysis and offered significant cultural insights that unpacked the nuances of participant responses.

While there are limits with generalizing beyond Marshallese living in Arkansas, this exploratory study provides important information on COFA migrants’ understanding and experience with the ACA. Qualitative methods allowed us to collect powerful and rich data on the lived experiences and policy interpretation from a segment of the population that is uniquely effected by the ACA and related policies. This study also provides a foundation for future research, policy change, education, and outreach programs.

### Recommendations for policy and practice

Several actions and policies of the U.S. federal government (COFA, ACA, PRWORA, nuclear testing, recruitment of Marshallese into the U.S. military, and the use of RMI land for military purposes) create challenges for COFA migrants living in the United States. While each of these actions and public policies are made at the federal level, states and local communities must now grapple with how to appropriately care for COFA migrants living within their borders. Consistent with our commitment to give voice to our CBPR stakeholders, we offer recommendations for policies and practices based upon the findings of this study.

At the federal level, policy action is needed to restore Medicaid for COFA migrants. COFA migrants were eligible for Medicaid when they agreed to the COFA, but were left out when PRWORA did not include COFA migrants in the category of “qualified immigrants” [46, 50]. A congressional delegation from Hawaii introduced bills in both the U.S. House of Representatives and the U.S. Senate in an attempt to amend Title IV of the PRWORA and restore Medicaid coverage for citizens of the Freely Associated States lawfully residing in the United States under the COFA. The House Bill (H.R. 2249) was referred to the House Committee on Commerce and Energy and the Senate Bill (S. 1301) was sent to the Senate Committee on Finance. Passage of this legislation would provide much-needed access to health care services for tens of thousands of COFA migrants.

In addition to legislative action, organizations can take steps to mitigate barriers. Local, state, and federal staff responsible for processing applications and enrollment for ACA need additional training on COFA migrants’ eligibility for coverage. One significant way to eliminate frustration and confusion at the local level is to employ more bilingual IPAs and navigators who are located in the communities where the majority of the Marshallese live. Bilingual fact sheets, both printed copies and on-line web documents, could also be provided to COFA migrants throughout the United States.

## Conclusion

While the ACA and Medicaid Expansion have reduced the uninsured rate nationally and in Arkansas [33], not everyone has benefited from this policy. Participants in this study recount inconsistent information and long wait times, along with reduced access to health care and medications. The Marshallese interpret the ACA, its requirements and penalties, and their lack of access to Medicaid and Medicaid Expansion as part of the broader relationship between the RMI and the United States rather than a discrete public policy. The COFA is described by the Marshallese as “jeṃ-jerā,” a deep friendship that binds our countries together in a relationship of commitment to care and support. “We created the COFA so that we can help America and so that America can help us.” While participants discuss their frustration with the current policies, rather than engaging in policy advocacy efforts, participants recount how the RMI and the Marshallese people have been good friends to the United States, and they appeal to the United States to honor their commitment to friendship with the RMI and the Marshallese people. The United States has the opportunity to honor our friendship with the Marshallese people by restoring Medicaid benefits, which would provide equal access to health care benefits.

## Abbreviations

ACA, Affordable Care Act; CBPR, Community-based participatory research; CHIP, Children’s Health Insurance Program; COFA, Compact of Free Association; DHS, Department of Human Services; IPA, In-person assistors; IRB, Institutional Review Board; PRWORA, Personal Responsibility and Work Opportunity Reconciliation Act; RMI, Republic of the Marshall Islands

## References

[1] Hixson L, Hepler BB, Kim MO. The Native Hawaiian and Other Pacific Islander Population: 2010. 2012. http://www.census.gov/prod/cen2010/briefs/c2010br-12.pdf. Accessed 14 Jan 2014.

[2] Shek D, Yamada S (2011). Health care for Micronesians and constitutional rights. Hawaii Med J.

[3] McElfish P (2013). Interview with Carmen Chong-Gum.

[4] Leonard C. Leaving the Islands: The long journey from the Marshall Islands to Northwest Arkansas. Little Rock, AR: Arkansas Democrat-Gazette; 2005. Accessed 15 May 2013.

[5] Jimeno R (2013). A Profile of the Marshallese Community in Arkansas.

[6] Riklon S, Alik W, Hixon A, Palafox N (2010). The “Compact impact” in Hawai‘i: focus on health care. Hawaii Med J.

[7] Barker H. Bravo for the Marshallese: Regaining control in a post-Nuclear, post-colonial world. 2nd ed. Belmont, CA: Cengage Learning; 2012.

[8] Cooke S (2010). In Mortal Hands: A cautionary history of the nuclear age.

[9] Pollock NJ (2002). Health transitions, fast and nasty: exposure to nuclear radiation. Developing Hum Resour Pacific.

[10] Guyer RL (2001). Radioactivity and rights: clashes at Bikini Atoll. Am J Public Health.

[11] Reddy R, Shehata C, Smith G, Maskarinec GG (2005). Characteristics of Marshallese with type 2 diabetes on Oahu: a pilot study to implement a community-based diabetic health improvement project. Calif J Health Promot.

[12] Gittelsohn J, Haberle H, Vastine A, Dyckman W, Palafox NA (2003). Macro- and microlevel processes affect food choice and nutritional status in the republic of the marshall islands. J Nutr.

[13] Hallgren E, McElfish P, Rubon-Chutaro J (2015). Barriers and opportunities: a community-based participatory research study of health beliefs related to diabetes in a US Marshallese community. Diabetes Educ.

[14] McElfish P, Hallgren E, Henry L, Ritok M, Rubon-Chutaro J, Kohler P (2016). Health beliefs of Marshallese regarding type 2 diabetes. Am J Health Behav.

[15] Donoho G, McElfish P, Avants R, Hallgren E (2015). A novel recruiting and surveying method: participatory research during a Pacific Islander community’s traditional cultural event. Gateways: Int J Community Res Engagement.

[16] McElfish P, Moore R, Woodring D (2016). Social ecology and diabetes self-management among Pacific Islanders in Arkansas. J Fam Med Dis Prev.

[17] McElfish P, Bridges M, Hudson J (2015). Family model of diabetes education with a Pacific Islander community. Diabetes Educ.

[18] McElfish P, Kohler P, Smith C (2015). Community-driven research agenda to reduce health disparities. Clin Transl Sci.

[19] Yamada S, Pobutsky A (2009). Micronesian migrant health issues in Hawaii: part 1: background, home island data, and clinical evidence. Calif J Health Promot.

[20] World Health Organization (2011). Marshall Islands.

[21] World Health Organization (2013). Global Tuberculosis Report 2013.

[22] World Health Organization (2013). Global policy report on the prevention and control of viral hepatitis in WHO Member States.

[23] Wong DC, Purcell RH, Rosen L (1979). Prevalence of antibody to hepatitis A and hepatitis B viruses in selected populations of the South Pacific. Am J Epidemiol.

[24] Woodall P, Scollard D, Rajan L (2011). Hansen Disease among Micronesian and Marshallese persons living in the United States. Emerg Infect Dis.

[25] Fischer G, Wang S, Ahring S (2009). An investigation of perinatal hepatitis B virus infections among a high risk population: the delivery hospital as a safety net. Pediatr Infect Dis J.

[26] Adams WH, Fields HA, Engle JR, Hadler SC (1986). Serologic markers for hepatitis B among Marshallese accidentally exposed to fallout radiation in 1954. Radiat Res.

[27] Brindle R, Eglin R, Parsons A, Hill A, Selkon J (1988). HTLV-1, HIV-1, hepatitis B and hepatitis delta in the Pacific and South-East Asia: a serological survey. Epidemiol Infect.

[28] Schempf AH, Mendola P, Hamilton BE, Hayes DK, Makuc DM (2010). Perinatal outcomes for Asian, Native Hawaiian, and Other Pacific Islander mothers of single and multiple race/ethnicity: California and Hawaii, 2003–2005. Am J Public Health.

[29] United States Supreme Court. King et al. v. Burwell, Secretary of Health and Human Services, et al. Washington, DC; 2015.

[30] Medicaid.gov. National Medicaid & CHIP Program Information. 2014; http://www.medicaid.gov/medicaid-chip-program-information/program-information/medicaid-and-chip-program-information.html. Accessed 19 Aug 2014.

[31] Medicaid.gov. Arkansas. 2014; http://www.medicaid.gov/Medicaid-CHIP-Program-Information/By-State/arkansas.html. Accessed 2 Jan 2015.

[32] Henry J. Kaiser Family Foundation. Medicaid Expansion in Arkansas. 2014; http://kff.org/medicaid/fact-sheet/medicaid-expansion-in-arkansas/. Accessed 3 Jan 2015.

[33] Witters D. Arkansas, Kentucky See Most Improvement in Uninsured Rates. Washington, D.C.; Gallup, Inc.; 2015. Accessed 12 May 2015.

[34] Asian and Pacific Islander American Health Forum. Key Facts on Medicaid Restoration for COFA Migrants. 2014. http://www.apiahf.org/programs/nhpi-affairs/community-capacity-building/medicaid-restoration-compact-free-association-mi-0. Accessed 28 March 2014.

[35] Asian and Pacific Islander American Health Forum. Medicaid Restoration for Compact of Free Association Migrants. 2014. http://www.apiahf.org/policy-and-advocacy/policy-priorities/health-care-access/medicaid-restoration-compact-free-associati. Accessed 28 April 2014.

[36] Geminiani V, Ostrowski D. Litigation in Federal and State Courts in Hawaii Preserves Critical Health Care for Micronesians. Chicago, IL: Sargeant Shriver National Center on Poverty Law; 2011.

[37] McElfish P, Hallgren E, Yamada S (2015). Effect of US health policies on health care access for Marshallese migrants. Am J Public Health.

[38] Cook SL. The Affordable Care Act: What does it mean to Arkansans? Little Rock, AR: Arkansas Insurance Department: 27; 2012. http://www.hbe.arkansas.gov/ACA.pdf. Accessed 20 May 2015.

[39] Kellams L (2013). Marshallese Families and the Affordable Care Act.

[40] United States Government Printing Office. 104th Congress Public Law 193. Washington, D.C.: 1996. http://www.gpo.gov/fdsys/pkg/PLAW-104publ193/html/PLAW-104publ193.htm. Accessed 13 April 2014.

[41] United States Court of Appeals for the Ninth Circuit. Appeal from the United States District Court for the District of Hawai’i Korab V. Fink. San Francisco: United States Court of Appeals for the Ninth Circuit; 2014. http://cdn.ca9.uscourts.gov/datastore/opinions/2014/04/01/11-15132.pdf. Accessed 12 Apr 2014.

[42] Creswell JW. Qualitative Inquiry and Research Design: Choosing among five approaches. Thousand Oaks, CA: 3rd ed. Sage; 2012.

[43] Tong A, Sainsbury P, Craig J (2007). Consolidated criteria for reporting qualitative research (COREQ): a 32-item checklist for interviews and focus groups. Int J Qual Health Care.

[44] Morgan DL, Gubrium JF, Holstein JA (2002). Focus Groups and social interaction. Handbook of interviewing research: context & methods.

[45] Pobutsky A, Krupitsky D, Yamada S (2009). Micronesian migrant health issues in Hawaii: part 2: an assessment of health, language and key social determinants of health. Calif J Health Promot.

[46] Hagiwara M, Yamada S, Tanaka W, Ostrowski D (2015). Litigation and community advocacy to ensure health access for Micronesian migrants in Hawai’i. J Health Care Poor Underserved.

[47] Center for Disease Control and Prevention. 2009 Marshallese Constitution Day Health Survey. Springdale AR: Centers for Disease Control and Prevention; 2009.

[48] Lincoln YS, Guba EG. Naturalistic Inquiry. Newbury Park, CA: SAGE Publications; 1985.

[49] Creswell JW, Miller DL (2000). Determining validity in qualitative inquiry. Theory Into Practice.

[50] Singer A, Kretsedemans P, Aparicio A (2004). Welfare Reform and Immigrants: A Policy Review. Immigrants, Welfare Reform, and the Poverty of Policy.

